# Adaptability of small brown planthopper to four rice cultivars using life table and population projection method

**DOI:** 10.1038/srep42399

**Published:** 2017-02-13

**Authors:** Xiao-Min Zheng, Yun-Li Tao, Hsin Chi, Fang-Hao Wan, Dong Chu

**Affiliations:** 1 Key Laboratory of Integrated Crop Pest Management of Shandong Province, College of Agronomy and Plant Protection, Qingdao Agricultural University, Qingdao 266109, China; 2 Department of Plant Production and Technologies, Faculty of Agricultural Sciences and Technologies, Ömer Halisdemir University, Turkey; 3 State Key Laboratory for Biology of Plant Diseases and Insect Pests, Institute of Plant Protection, Chinese Academy of Agricultural Sciences (CAAS), Beijing 100081, China

## Abstract

In this study, we evaluated the adaptability of the small brown planthopper (SBPH), *Laodelphax striatellus* (Hemiptera: Delphacidae) to four rice cultivars including Shengdao13 (SD13), Shengdao14 (SD14), Shengdao15 (SD15), and Zixiangnuo (ZXN) using the age-stage, two-sex life table with a simplified method for recording egg production (i.e., every five days vs. daily). The intrinsic rate of increase (*r*) of the SBPH was the highest (0.1067 d^−1^) on cultivar SD15, which was similar to the rate on SD14 (0.1029 d^−1^), but was significantly higher than that occurring on ZXN (0.0897 d^−1^) and SD13 (0.0802 d^−1^). The differences of the finite rate of increase (*λ*) on the four rice cultivars were consistent with the *r* values. Population projection predicted an explosive population growth of the SBPH occurring in a relatively short time when reared on SD14 and SD15. These findings demonstrated that the SBPH can successfully survive on the four rice cultivars, although there were varying host adaptabilities.

Life table construction is a comprehensive method for summarizing the survival and reproductive potential of a population^1^. This method, which has been widely applied in the measurement of population parameters of insects under varied ecological conditions, is helpful to the development of integrated control strategies^2^,^3^,^4^. The age-stage, two-sex life table is especially useful in this regard because it enables the users to not only precisely describe the population characteristics but also allows the stage differentiation and both sexes to be taken into consideration. This life table has recently gained wide acceptance and has been extensively adopted to determine the effects of ecological factors including host plants^5^,^6^,^7^, humidity^4^, temperature^8^,^9^, and pesticides^10^ on growth potential of insect populations. One of the major drawbacks, however, of using this and other life tables has been the amount of time and effort involved in the collection of raw data. This is particularly time-consuming during the oviposition period when it is necessary to count the eggs laid by each adult female daily. In our recent study^11^, we demonstrated that a simplified recording method (i.e., at intervals of several days) during the egg production period could be adopted for the age-stage, two-sex life table. Using this technique will allow the researchers to save time and labor and to avoid daily disturbance of the adults – a disruption that has been recognized and mentioned by previous researchers^12^. Because this improved method has been shown to not have a significant impact on key population parameters^11^, adopting this simplified procedure to collect data for construction of life tables will be helpful in applying life table methods in assessing population parameters and growth potential of populations.

The small brown planthopper (SBPH), *Laodelphax striatellus* (Hemiptera: Delphacidae) which is one of the most important insect pests on rice, *Oryza sativa* L., acts as the major vector of rice stripe virus (RSV)^13^,^14^,^15^. Due to its high dispersal ability, the planthopper has become widely distributed throughout much of eastern Asia including China, Japan, and Korea. Because of this dispersal ability and migrating behavior, managing the SBPH in rice crops has been especially difficult^13^. In attempting to avoid the many negative effects associated with repeated insecticide applications including pesticide resistance, chemical residues, reduction in the abundance and/or number of natural enemies, etc., the use of pest-resistant cultivars has become an economical, effective, and environment-friendly control strategy for managing agricultural pest insects^16^,^17^. Because of its versatility, the life table method is an ideal tool for screening the suitability of pest-resistant rice cultivars in order to comprehensively evaluate the adaptability of insects to the cultivars.

To evaluate the resistance effectiveness of different rice cultivars to the SBPH, we conducted demographic comparisons of the planthopper on four rice cultivars, Shengdao13 (abbreviated as SD13), Shengdao14 (abbreviated as SD14), Shengdao15 (abbreviated as SD15), and Zixiangnuo (abbreviated as ZXN), using the age-stage, two-sex life table with a simplified recording method (i.e. recording egg production every five-days instead of daily). The results will help in the selection of resistant rice cultivars, provide useful data for future rice breeding programs, and contribute to the effective management of the SBPH. This study also confirms the feasibility of using a simplified, but effective, means of recording egg production in life table studies. Using the procedure in future demographic research will save considerable time and effort.

## Results

### Population parameters of the SBPH on the four rice cultivars

There were no significant differences between the SD14 and SD15 cultivars in any of the basic statistics, including preadult developmental time, preadult survival rate, female and male longevity and mean fecundity (*F*). Among the basic statistics, the SD14 parameters were significantly different from the corresponding values found in SD13 and ZXN except egg mortality. The values from SD15 were significantly different from these on SD13 with the exception of egg and nymph duration and were significantly different from these on ZXN with the exception of egg and nymph mortality, nymph duration and female longevity (Table 1).

The values for *N*_*f*_ (number of emerged female adults) and *N* (number of eggs at the beginning) were: 56 and 141 on SD13. The total preadult survival of the SBPH was 50%. Out of the initial 208 eggs laid on SD14, 52 eventually developed into female adults and 21 into male adults. The preadult survival on this cultivar was 35%. On SD 15, there were 181 eggs and a preadult survival of only 33%, while on ZXN, the preadult survival was 45% out of 175 eggs (Table 1). There were more adult females than males produced in each of the four rice cultivars.

**Figure 1:**
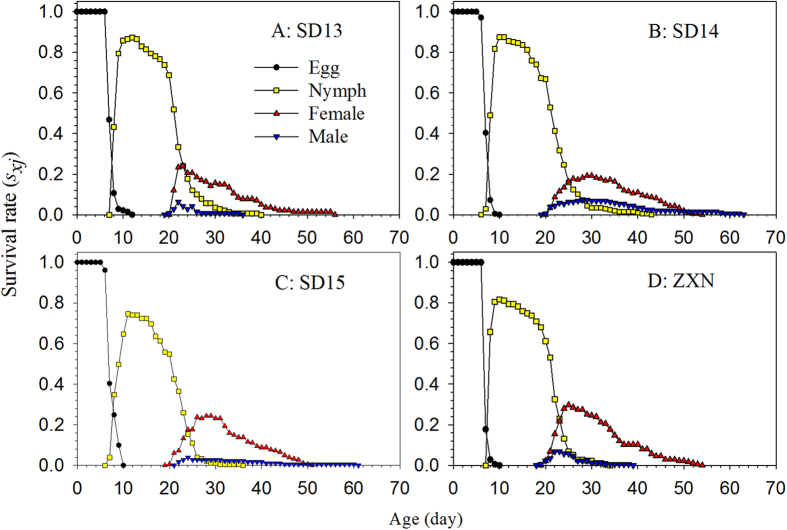
Age-stage specific survival rate (*S*_*xj*_) of the SBPH on four rice cultivars.

**Figure 2:**
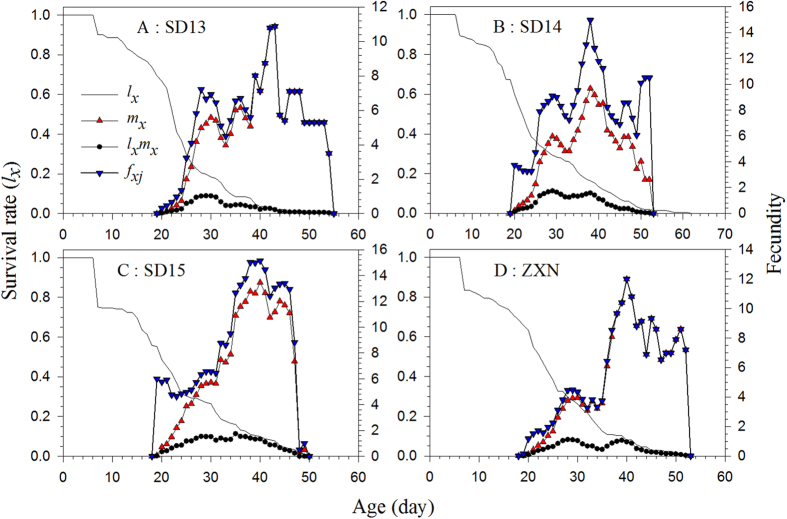
Age-specific survival rate (*l*_*x*_), female age-specific fecundity (*f*_*xj*_), age-specific fecundity of the total population (*m*_*x*_), and age-specific maternity (*l*_*x*_*m*_*x*_) of the SBPH on four different rice cultivars.

The survivorship and stage differentiation of individuals reared on the four rice cultivars can be observed in the age-stage survival rate (*s*_*xj*_). The female and male curves emerged at age 19d and 18d, respectively, for both the SD13 and SD14 cultivars (Fig. 1A and B); in contrast, females appeared at 18d for the SD15 and ZXN cultivars, while males first appeared at 20d on SD15 and 17d on ZXN, respectively (Fig. 1C and D). The female age-stage specific fecundity (*f*_*xj*_) and age-specific fecundity (*m*_*x*_) on the SD13 and SD14 cultivars began at 19d (Fig. 2A and B), only 1d later than on the SD15 and ZXN cultivars (at 18d) (Fig. 2C and D).

The intrinsic rate of increase (*r*) of the SBPH was the highest (0.1067 d^−1^) on cultivar SD15, which was similar to the rate on SD14 (0.1029 d^−1^), but was significantly higher than that occurring on ZXN (0.0897 d^−1^) and SD13 (0.0802 d^−1^). The differences of the finite rate of increase (*λ*) on the four rice cultivars were consistent with the *r* values. Although there were no significant differences found between the net reproductive rates (*R*_0_) of the SBPH on the SD15, SD14 and ZXN cultivars (30.33, 28.81, and 18.55 eggs/individual, respectively), the first two rates were significantly higher than the SD13 rate (13.06) (*P* < 0.05). There were no significant differences among mean generation times (*T*) of the SBPH on the four rice cultivars (Table 2). The relationship among *F, R*_0_, *N*_*f*_, and *N* in all treatments were consistent with the proof given by Chi^29^.

The age-stage specific fecundity (*f*_*xj*_), i.e., the mean number of offspring produced by a female adult at age *x*, is shown in Fig. 2. The highest *f*_*xj*_ peaks and age-specific fecundity of the total population (*m*_*x*_) occurred at approximately age 40 d. However, due to the lower survival rate at this age, the highest age-specific maternity (*l*_*x*_*m*_*x*_) occurred at approximately age 30 d. The highest peaks for *f*_*xj*_, *m*_*x*_, and *l*_*x*_*m*_*x*_were observed in the SBPH population reared on cultivar SD15.

**Figure 3:**
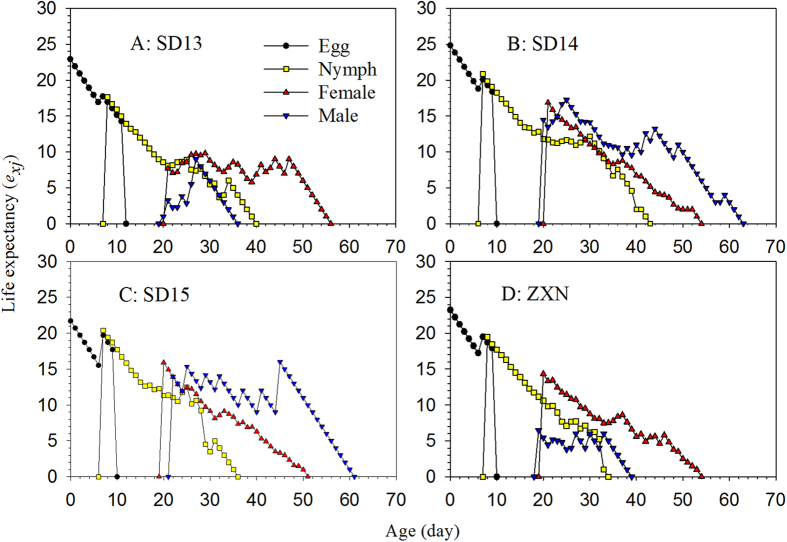
Age-stage specific life expectancy (*e*_*xj*_) of the SBPH on four different rice cultivars.

**Figure 4:**
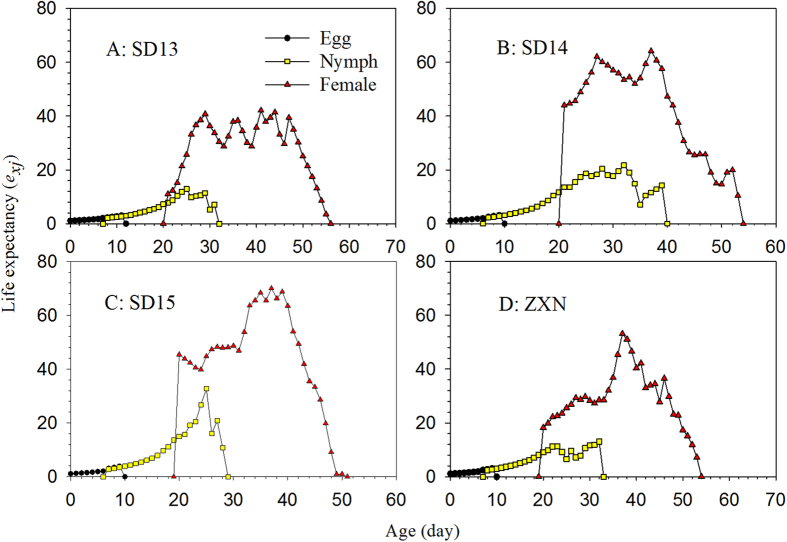
Reproductive value (*v*_*xj*_) of the SBPH on four different rice cultivars.

The life expectancies of the SPBH at age zero (*e*_01_) (22.94, 24.87, 21.72, and 23.22 d on cultivars SD13, SD14, SD15, and ZXN, respectively) were not significantly different (Fig. 3). These values were exactly the same mean longevity of all individuals used in the life table study. At age zero, the reproductive values (*v*_01_) were the same as the finite rates on the four rice cultivars, i.e., 1.0835 d^−1^ on SD13, 1.1084 d^−1^ on SD14, 1.1126 d^−1^ on SD13, and 1.0938 d^−1^ on ZXN (Fig. 4). The values for the reproductive value, *v*_*xj*_, on cultivars SD13 and ZXN increased to 11.03 d^−1^at 20 d and 18.26 d^−1^ at 19 d when the female adults emerged. When reared on the SD14 and SD15 cultivars, however, the *v*_*xj*_ values jumped to 44.05 d^−1^ and 45.40 d^−1^ at age 20 d when the female adults emerged (Fig. 4).

### Population projection of the SBPH on the four rice cultivars

**Figure 5:**
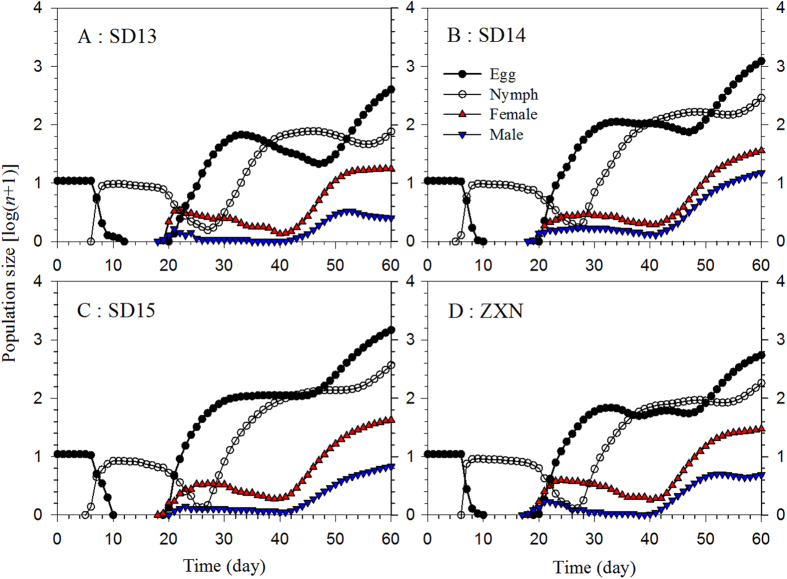
Population projection of the SBPH on four different rice cultivars. An initial population of 10 eggs was used in each projection.

The population size of the different stages simulated from an initial population of 10 eggs using the TIMING-MSChart program is shown in Fig. 5. The population projection showed that the growth of the SBPH individuals was the slowest on cultivar SD13, and the fastest on cultivar SD15. After 60 days on SD13, there were 476 nymphs, 16 female and 2 male adults; while on SD15 there were 1845 nymphs, 42 female and 6 male adults. The stage curves showed the trend and emergence time of different stages.

## Discussion

The use of pest-resistant host plants is regarded as one of the most effective means of decreasing a pest population size and limiting the use of pesticides in agricultural ecosystems in IPM (integrated pest management) programs^18^. The life table method can be used to evaluate the host adaptation of insect pests to their host plant. Variations in host plant suitability can affect life table parameters of herbivorous insects. Use of the age-stage, two-sex life table method in this study graphically demonstrated the adaptation of the SBPH to the four different rice cultivars, SD13, SD14, SD15, and ZXN, which are the primary Japonica rice cultivars grown in Shandong Province.

The intrinsic rate of increase (*r*) is a key parameter in measuring a plant’s resistance to insects because it reflects the physiological qualities of an animal in relation to its capacity to increase^19^. It is, therefore, one of the most appropriate indices for evaluating the performance of an insect on host plants as well as the host plant’s suitability to herbivores^20^,^21^. Comparisons of the *r* or *R*_0_ values between different populations can often provide considerable insight beyond that obtainable from these individual life-history parameters based on independent analysis of the values^22^. In this study, comparative analysis of the *r, λ*, and *R*_0_ values indicated that SD15 is the most suitable cultivar for the development of the SBPH although the preadult survival rate was low. The low preadult survival rate of SD 15 was mainly due to the low egg hatch rate.

Population projections based on an age-stage, two-sex life table can predict changes of stage structure during population growth^6^,^23^. Our projections demonstrated that the growth of the SBPH population would be the fastest on cultivar SD15 and the slowest on SD13 (Fig. 5). Projections are also useful in providing valuable information on the trends and emergence timing of not only the preadult stages, but the female and male adult emergences as well. This information is also relevant for implementing pest control schedules using more conventional means of control including pesticides or physical control (e.g., light traps). The different fitness levels of the SBPH on new and existing rice cultivars can be used as indicators in screening resistance genes in rice breeding programs.

In previous studies, fecundity was determined by daily observations on the deposited eggs until the female adults died^4^,^8^,^24^. In this study, we used a simplified recording method of recording egg production where the eggs laid by females were counted every five days instead of daily. Our previous study^11^ indicated that no significant differences occurred in the population parameters (intrinsic rate of increase (*r*), finite rate of increase (*λ*), net reproductive rate (*R*_0_), mean generation time (*T*), preadult duration, fecundity, female longevity and male longevity) obtained through daily sampling versus the interval-grouped data, except in the adult preoviposition period (APOP) and oviposition days. In this study, “five days” was considered to be an appropriate sampling interval because the eggs generally begin to hatch on the sixth day when kept at a suitable temperature *e.g*., 28 °C^25^.

Hu *et al*.^12^ pointed out that the number of eggs laid by the planthopper, *Nilaparvata lugens* (Stål) (Hemiptera: Delphacidae), on the rice cultivar “Taichung Native 1” (*O. sativa indica* TN1) decreased significantly compared to the number found by Kisimoto^26^. The difference may be due to the daily replacement of rice plants disturbing the feeding and oviposition behavior of the female planthoppers^12^. The simplified recording method used in this study, will not only save considerable time and labor, but would also significantly reduce the amount of disruption to the insect’s behavior caused by the replacement of host plants. If the procedure becomes more widely adopted in life table studies, it may help to promote further applications of the life table method and thereby benefit future ecological research. This study is the first to apply a simplified recording method in an age-stage, two-sex life table study to evaluate the demographic characteristics of an insect population. Life table data compilation using simplified recording methods can efficiently provide a comprehensive description of the survival, development, and reproduction of a cohort of individuals.

## Materials and Methods

### Insects

The SBPH, *L. striatellus*, was collected from Liaoning Province, China in October, 2014 and reared on rice seedlings (SD13) in glass beakers (14 cm in diameter, 19 cm in height) in a controlled climate chamber (27 ± 1 °C, a photoperiod L: D = 16: 8 h, and 60 ± 5% RH). The glass beakers containing 2–3 cm high rice seedlings were covered with a piece of mesh cloth for ventilation after the insects were added. The newly grown seedlings used for rearing the *L. striatellus* population were replaced every 10–15 days.

### Plants

Four rice cultivars, SD13, SD14, SD15, and ZXN, were used in the study. The cultivar ZXN was collected from Dongying, Shandong Province, China, while SD13, SD14 and SD15 were collected from Jinan, Shandong Province, China. All seedlings were cultivated in plastic pots with nutrient soil (organic matter ≥35%; pH = 5.5~6.5) under controlled conditions in a screen house (27 ± 1 °C, a L: D = 16: 8 h photoperiod, and 60 ± 5% RH).

### Experiments

Using the method described by Duan *et al*.^27^, ten approximately 5 cm high seedlings (1–2 leaves) of one of the rice cultivars were placed in a beaker, and the process repeated for each of the four cultivars. Ten gravid female adults, randomly selected from the rearing population, were transferred into each beaker^12^. After 24 h, all adult females were removed and each of the seedlings was transferred into a glass tube (2 cm in diameter, 18 cm in height). These glass tubes were then enclosed with a piece of mesh cloth (200 per square centimeter) for ventilation, and water was added along the tube wall when necessary. Experiments were conducted at 27 ± 1 °C, L: D = 16: 8 h photoperiod, and 60 ± 5% RH. The seedlings in the glass tubes were checked daily for newly hatched nymphs beginning at the 5^th^ day, which corresponded to the day prior to egg hatching. When no newly hatched nymphs were found after a 72 hr. period, the seedlings were removed and dissected under a stereomicroscope (Nikon SMZ 745T) to check for inviable eggs. The numbers of hatched and unhatched eggs were counted for each of the seedlings. The total numbers of eggs found that were used for the life table study were 141, 208, 181, and 175 eggs on the SD13, SD14, SD15, and ZXN cultivars, respectively. Each of the newly hatched 1st instar nymphs was transferred into a separate glass tube containing a new rice seedling, and observed daily for survival. Newly emerged male and female adults resulting from the treatment were paired and transferred to a new glass tube containing three rice plants for oviposition. If there were surplus individuals of one sex, they were paired with young adults of the opposite sex recruited from the mass-reared colony for mating. Because the recruited adults were added solely for mating purpose, they were excluded from analysis. The adult SBPH individuals were checked daily for survival. To record egg production, each pair of the SBPHs was transferred into a new glass tube containing three seedlings every five days until the death of all individuals. The mean daily fecundity during the 5-days period was determined by dissecting the three seedlings under a stereomicroscope as described above. All of the newly emerged adult females were paired with adult males and then used to evaluate fecundity (Table 1). If a male died before the female, a new male was added for mating purposes, but only the longevity and fecundity of the female was recorded. If the female died before the male, we added a new female but only recorded the longevity of the male.

### Statistical analysis

The life history raw data of all the SBPH individuals were analyzed based on the age-stage, two-sex life table^28^,^29^ using the computer program TWOSEX-MSChart. The program is available for no cost at: http://140.120.197.173/Ecology/download/TWOSEX-MSChart.rar. The population parameters that were calculated included the age-stage specific survival rate (*s*_*xj*_, the probability that a newborn will survive to age *x* and stage *j*), the age-stage specific fecundity (*f*_*xj*_, the mean number of offspring produced by a female of age *x*), the age-specific survival rate (*l*_*x*_, the probability of a newly laid egg surviving to age *x*), the age-specific fecundity (*m*_*x*_, the mean fecundity of individuals at age *x*), the age-stage life expectancy (*e*_*xj*_, the length of time that an individual of age *x* and stage *j* is expected to live), and the reproductive value (*v*_*xj*_, the contribution of an individual to the future population).

In the age-stage, two-sex life table^28^, *l*_*x*_ and *m*_*x*_ are calculated as:


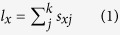



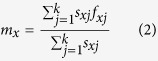


where *k* is the number of stages. The net reproductive rate (*R*_0_) is calculated as:


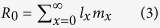


The intrinsic rate of increase (*r*) is calculated as:


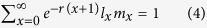


with age indexed from 0^30^. The finite rate of increase (*λ*) is calculated as *λ* = *e*^*r*^. The mean generation time *T* is defined as the length of time that a population needs to increase *R*_0_ fold of its size (i.e., *e*^*rT*^ = *R*_0_ or *λ*^*T*^ = *R*_0_) at a stable age distribution, and is calculated as:


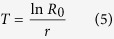


According to Chi and Su, the life expectancy *e*_*xj*_is calculated as:


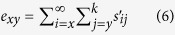


where *s*^*′*^_*ij*_is the probability that an individual of age *x* and stage *y* will survive to age *i* and stage *j* and is calculated by assuming *s*^*′*^_*xy*_ = 1^31^. The reproductive value (*v*_*xj*_) is calculated according to Huang and Chi^32^ and Tuan *et al*.^8^ as:


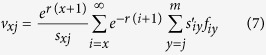


The standard errors of all life table parameters including *r, λ, R*_0_, *T*, adult longevity, and fecundity were estimated by the bootstrap procedure with 100,000 resampling. A paired bootstrap test was used to detect the difference among cultivars based on the confidence interval of differences^33^,^34^,^35^. SigmaPlot v. 12.0 software was used to create the graphs.

### Population projection

We projected the population growth to illustrate the predicted population size and age-stage structure of the SBPH by using survival rate and fecundity data^23^,^28^ using the TIMING-MSChart program^36^. The TIMING-MSChart program is also available at: http://140.120.197.173/ ecology/Download/TIMING-MSChart.rar.

## Additional Information

**How to cite this article:** Zheng, X.-M. *et al*. Adaptability of small brown planthopper to four rice cultivars using life table and population projection method. *Sci. Rep.* **7**, 42399; doi: 10.1038/srep42399 (2017).

**Publisher's note:** Springer Nature remains neutral with regard to jurisdictional claims in published maps and institutional affiliations.

## Figures and Tables

**Table 1:** Preadult developmental time, longevity, and mean fecundity (*F*) of the SBPH on four rice cultivars.

Basic statistic	Rice cultivar							
	*n*	SD13	*n*	SD14	*n*	SD15	*n*	ZXN
Egg duration	127	7.71 ± 0.08a	186	7.51 ± 0.05b	136	7.95 ± 0.10a	146	7.25 ± 0.04c
Egg mortality	141	0.099 ± 0.025b	208	0.106 ± 0.021b	181	0.249 ± 0.032a	175	0.166 ± 0.028ab
Nymph duration	71	14.15 ± 0.27b	73	16.04 ± 0.41a	60	14.78 ± 0.28b	78	14.59 ± 0.25b
Nymph mortality	127	0.397 ± 0.041b	186	0.543 ± 0.034a	136	0.420 ± 0.037b	175	0.389 ± 0.037b
Preadult duration (d)	71	21.89 ± 0.28b	73	23.40 ± 0.43a	60	22.85 ± 0.28a	78	21.79 ± 0.25b
Preadult survival rate	141	0.50 ± 0.04a	208	0.35 ± 0.03bc	181	0.33 ± 0.03c	175	0.45 ± 0.04ab
Female longevity (d)	56	29.82 ± 1.09c	52	38.08 ± 1.12a	52	35.77 ± 1.04ab	61	34.61 ± 0.99b
Male longevity (d)	15	24.07 ± 0.88b	21	36.86 ± 2.61a	8	35.88 ± 4.37a	17	25.82 ± 1.05b
Fecundity (*F*) (eggs per female)	56	32.89 ± 7.21b	52	115.23 ± 12.55a	52	105.56 ± 13.63a	61	53.21 ± 8.11b

Data followed by the same lower-case letter in the same row were not significantly different based on a paired bootstrap test at the 5% significance level.

**Table 2:** Means and standard errors of the intrinsic rate of increase (*r*), finite rate (*λ*), net reproductive rate (*R*_0_), and mean generation time (*T*) of the SBPH reared on four rice cultivars.

Parameters	Rice cultivars			
	SD13	SD14	SD15	ZXN
*r* (d^−1^)	0.0802 ± 0.0075c	0.1029 ± 0.0053ab	0.1067 ± 0.0056a	0.0897 ± 0.0051bc
*λ* (d^−1^)	1.0835 ± 0.0081c	1.1084 ± 0.0059ab	1.1126 ± 0.0062a	1.0938 ± 0.0056bc
*R*_0_ (offspring/individual)	13.06 ± 3.16b	28.81 ± 4.65a	30.33 ± 5.27a	18.55 ± 3.41ab
*T* (d)	32.05 ± 1.17a	32.65 ± 0.65a	31.99 ± 0.73a	32.57 ± 0.74a

Standard errors were estimated using 100,000 bootstrap resampling. A paired bootstrap test was used to detect differences between treatments.
